# Perspectives on the Intersection of Electronic Health Records and Health Care Team Communication, Function, and Well-being

**DOI:** 10.1001/jamanetworkopen.2023.13178

**Published:** 2023-05-12

**Authors:** Alexis Amano, Cati G. Brown-Johnson, Marcy Winget, Amrita Sinha, Shreya Shah, Christine A. Sinsky, Christopher Sharp, Tait Shanafelt, Kelley Skeff

**Affiliations:** 1 Department of Medicine, Division of Primary Care and Population Health, Stanford University School of Medicine, Stanford, California; 2 Divisions of Medical Critical Care and Clinical Informatics, Harvard Medical School, Boston, Massachusetts; 3 American Medical Association, Chicago, Illinois; 4 Division of Hematology and General Internal Medicine, Department of Medicine, Stanford University School of Medicine, Stanford, California; 5 WellMD Center, Stanford University School of Medicine, Stanford, California; 6 Department of Health Policy and Management, Fielding School of Public Health, University of California. Los Angeles

## Abstract

**Importance:**

Understanding of the interplay between the electronic health record (EHR), health care team relations, and physician well-being is currently lacking. Approaches to cultivate interpersonal interactions may be necessary to complement advancements in health information technology with high-quality team function.

**Objective:**

To examine ways in which the EHR, health care team functioning, and physician well-being intersect and interact.

**Design, Setting, and Participants:**

Secondary qualitative analysis of semistructured interview data from 2 studies used keyword-in-context approaches to identify excerpts related to teams. Thematic analysis was conducted using pattern coding, then organized using the relationship-centered organization model. Two health care organizations in California from March 16 to October 13, 2017, and February 28 to April 21, 2022, participated, with respondents including attending and resident physicians.

**Main Outcome and Measures:**

Across data sets, themes centered around the interactions between the EHR, health care team functioning, and physician well-being. The first study data focused on EHR-related distressing events and their role in attending physician and resident physician emotions and actions. The second study focused on EHR use and daily EHR irritants.

**Results:**

The 73 respondents included attending physicians (53 [73%]) and resident physicians (20 [27%]). Demographic data were not collected. Participants worked in ambulatory specialties (33 [45%]), hospital medicine (10 [14%]), and surgery (10 [14%]). The EHR was reported to be the dominant communication modality among all teams. Interviewees indicated that the EHR facilitates task-related communication and is well suited to completing simple, uncomplicated tasks. However, EHR-based communication limited the rich communication and social connection required for building relationships and navigating conflict. The EHR was found to negatively impact team function by promoting disagreement and introducing areas of conflict into team relationships related to medical-legal pressures, role confusion, and undefined norms around EHR-related communication. In addition, interviewees expressed that physician EHR-related distress affects interactions within the team, eroding team well-being.

**Conclusions and Relevance:**

In this study, the EHR supported task-oriented and efficient communication among team members to get work done and care for patients; however, participants felt that the technology shifts attention away from the human needs of the care team that are necessary for developing relationships, building trust, and resolving conflicts. Interventions to cultivate interpersonal interactions and team function are necessary to complement the efficiency benefits of health information technology.

## Key Points

**Question:**

How do electronic health records (EHRs), team functioning and communication, and clinician well-being intersect from the point of view of physicians?

**Findings:**

In this qualitative study comprising 73 attending and resident physicians, the EHR was reported to be the dominant communication modality among teams, facilitating straightforward and task-related communication but limiting rich and social communication. The EHR was reported to negatively affect team function and team well-being.

**Meaning:**

The findings of this study suggest that positive aspects of EHR use support task-oriented and efficient communication among team members; however, the technology shifts attention away from the human needs of the care team, and interventions to cultivate interpersonal interactions and team function are necessary to complement the efficiency benefits of health information technology.

## Teaser

This qualitative study examines the perspectives of attending physicians and resident physicians on the use of electronic health records.

## Introduction

Health care team functioning and communication constitute major elements of health care workplace culture and are intertwined with burnout and work satisfaction.^1,2,3,4^ Not only do team function and communication play a role in clinician well-being, but these dimensions of organizational behavior also affect institutions’ financial, clinical, and operational performance.^3,5,6^

The electronic health record (EHR) also affects communication, clinician well-being, and teamwork (eFigure 1 in Supplement 1). Although use of the EHR supports clinical work and patient care, the ways it has altered care delivery are also major sources of physician distress, increasing administrative tasks, increasing time spent on work outside of scheduled hours, and decreasing physician time for patient care.^7,8,9,10,11,12,13,14,15,16,17^ A previous qualitative study by several of the present authors indicated that EHR requirements often take precedence over other physician core competencies, disrupting professional conduct and causing distress to physicians and team members.^18^ These results are also consistent with national longitudinal studies of increasing burnout among US physicians and nurses and decreasing physician work hours in parallel with increasing EHR integration in physician workflow.^19,20,21^

Despite the important roles that team dynamics and EHR play in physician well-being, the association between EHR and team relational dimensions is not well understood. Early work in this area has uncovered conflicting findings: EHR and other technology-based communication strategies may improve, hurt, or have no impact on team dynamics (eg, EHR messaging may enhance team communication,^11^ but EHR adversely affects team dynamics and may challenge diagnostic processes^22^).^6,11,22,23,24^

The relationship-centered organization (RCO) model posits that relationships are central in health care organizations and identifies 7 key relationship qualities. As such, the RCO is well suited to frame analysis of the relationships between the EHR and team relational dimensions. Earlier studies have used this model to explore the EHR and physician-patient relationships, finding that the EHR has the potential to facilitate (eg, use of asynchronous communication) or impede (eg, limiting face-to-face interactions) relationships.^5,7,25,26,27^ To our knowledge, the RCO model has not yet been used to evaluate the association of the EHR and clinical team relationships. Qualitative research is well suited to assess the nuanced ways in which the EHR and team function may interact. The goal of our qualitative analysis of physician interviews was to understand the ways in which EHR, team functioning and communication, and well-being intersect from the point of view of physicians, using the RCO model.

## Methods

This analysis includes 2 data sets. The initial data set focused on experiences of physician distress related to the EHR.^18^ After identifying that daily irritants were a major component of clinician distress but were less likely to be reported, a second qualitative study explored this further (eMethods in Supplement 1). The Consolidated Criteria for Reporting Qualitative Research (COREQ) reporting guideline was used to inform reporting of the study findings.^28^ All aspects of both studies were approved by the Stanford Institutional Review Board. Participants in both studies provided verbal consent prior to interviews.

In the initial study, interviews (n = 50) with faculty and residents in inpatient and outpatient specialties were conducted at an academic medical center and community hospital in Northern California. Phone interviews were completed from March 16 to October 13, 2017 (eTable 1 in Supplement 1).^18^ In the follow-up study, interviews (n = 23) of ambulatory care physicians were conducted at an academic medical center in Northern California from February 28 to April 21, 2022 (eTable 2 in Supplement 1).

We used key word-in-context approaches to identify excerpts related to teams within our 265 000-word data set.^29,30^ To identify key words, we used a multistep process: first, a qualitative researcher (A.A.) selected 4 representative excerpts from the data set. Those excerpts were reviewed independently by 3 of us for team-related key words (C.G.B.J. [qualitative research expert], M.W. [epidemiologist], and K.S. [medical education expert]). The qualitative researcher (A.A.) then compiled and categorized the inclusive list of key words based on topic area and, using this inclusive list, searched the data set for each term with qualitative software (NVivo 12, QSR International). The qualitative researcher then examined each identified key word in the context of the data set to understand its meaning and usage in relation to teams.^29,30^ Terms that did not return a relevant excerpt in a random selection of 5 excerpts were discarded. Terms were added to the list if they appeared in 3 different team-related passages. Relevant passages (254) were reviewed by the same qualitative researcher and organized via pattern coding—a coding method in which data are organized based on emerging patterns or themes.^31^ Early themes were reviewed by all of us. The final synthesis stage used RCO as an organizing model (Table 1).^5^ Themes were mapped to RCO by the same qualitative researcher (A.A.) and then discussed (A.A. and C.G.B.J.).

**Table 1.** Characteristics of Interview Participants

Characteristic	Participants, No. (%) (N=73)
Career stage	
Resident physician	20 (27)
Attending physician	53 (73)
Interviewee organization	
SHC	41 (56)
UHA	7 (10)
CPMC	25 (34)
Specialty area	
Ambulatory care	33 (45)
Hospital medicine	10 (14)
Surgery	10 (14)
Trainee	20 (27)

Abbreviations: CPMC, California Pacific Medical Center; SHC, Stanford Health Care; UHA, University HealthCare Alliance.

## Results

The 73 respondents included attending (53 [73%]) and resident (20 [27%]) physicians (Table 1). Demographic data were not collected. Most worked in ambulatory care specialties (33 [45%]); a subset worked in hospital medicine (10 [14%]) and surgery (10 [14%]).

Our analysis uncovered 3 major areas of EHR interaction with teams: (1) team communication, (2) team function, and (3) team member well-being. These themes suggest that current EHR use is affecting the actualization of 6 of the 7 key relationship qualities called for by RCO: team communication (mix of rich and lean communication and mix of social and task-oriented communication), team function (trust, mutual respect, mindfulness, and heedful interrelating), and team member well-being (heedful interrelating) (Table 2). We use lean terminology because that is enlisted in the RCO model; lean in this context is not associated with the manufacturing concept of lean. The seventh principle, diversity of mental models, which refers to valuing multiple and varied ways of thinking,^5^ was not found in our data.

**Table 2.** The EHR in Teams and the Actualization of RCO Constructs

Theme and subtheme	RCO construct^a^	Exemplar quotation
**EHR and communication**		
EHR supports task-oriented communication and hinders social connection	Mix of social and task-oriented communication^b^	“I suppose like being able to coordinate care maybe a little bit better with other providers that use the EHR or need to be able to access records from outside that may be connected. That has been helpful for me to provide better care for patients.” (physician 8; 2018)
		“So, it’s [completing EHR documentation] taking away from, as I was saying, just interaction with your patients, with the other professional staff… we’re not talking to each other in person. So, it takes away from that both in patient care and collegially.” (physician 2; 2018).
EHR supports lean communication and hinders rich communication	Mix of rich and lean communication^c^	“… I can quickly answer messages about, ‘Does this person need an appointment? Should this person get labs?’ All those type of things I can answer on my phone.” (physician 18; 2022)
		“… when there's disagreement, not infrequently people take that easy way and just put a note in and explain like, oh, what they think the other party did wrong… they don't actually talk to the other party to resolve the issue.” (physician 2; 2018)
**EHR and team function**		
Medical-legal characteristics of EHR can harm provider trust	Trust^d^	“… there’s a lot of burden to like use the EMR both as like the portal of communication… I think there’s also like this underlying, also sort of like a tone of like litigation… there’s a lot of stress around the timing of the documentation, how you word the documentation, and I think that’s also like something that’s a player in all of this…. It’s very stressful.” (physician 12; 2018)
EHR role confusion challenges mutual respect between physicians and other clinicians	Mutual respect^e^	“I feel like it was much different maybe with like before the EMR because the nurses did not just like hound you to put in these orders that are just actually routine for every single patient, right?… Yeah, it kind of like stresses our relationship with the nurses because you are like, ‘I told you I would put those orders in when I got a chance. Like, I have been with a patient since I last saw you. Like, no I have not put any orders.’ And, to us, like the orders are very clearly a second to taking care of patients. Right? So, if we are with a patient, like I am not sitting on the computer putting in orders for a different patient, but like the nurses just like do not see that. And it is a burden, and it is a stressor to our relationship with them…” (physician 19; 2018)
Lack of norms around EHR communication challenges mindfulness and heedful interrelating	Mindfulness^f^	“… we get calls, ‘Oh, you need this refill,’ or, ‘Oh, you need to clarify this order.’ I say, ‘Fine. I'm going to give you a verbal order.’ ‘Well, I can't take a verbal order. You can't put it in Epic.’ ‘Well, I'm not going to give you a verbal order, because I'm in the middle of a bike ride in the mountains and screw you.’ And the conversation goes south from there, and someone writes you up and says, ‘Well, this person wasn't Epic compliant, because I can't take a verbal order unless it's an absolute emergency, and it really was just for Tylenol’… And then I got to sit in front of a committee. I'm so sorry for not stopping, riding home, logging onto my computer, and co-signing a Tylenol order for Nurse Ratchet. So, I exaggerate little bit.” (physician 21; 2022)
	Heedful interrelating^g^	
**EHR and team member well-being**		
EHR limitations on sources of physician joy	Heedful interrelating^g^	“I think it's frustration and sort of, I mean, sometimes it's sadness because I feel like the joy that I get from practicing medicine is in spending time with patients and, you know, actually thinking about the medicine, but a lot of my time is taken up by writing notes and being on the computer, and so I think that they're contradictory in many ways. So, yeah, I think probably frustration and like some element of depression.” (physician 1; 2018)
Physician frustration over EHR design flaws impacts other interactions	Heedful interrelating^g^	“I think for me overall, you know, there is kind of like a general level of stress and like the more stressed I am, the less enjoyment I get out of being in the hospital and probably less pleasant to be around…” (physician 25; 2018)

Abbreviations: EHR, electronic health record; EMR, electronic medical record; RCO, relationship-centered organization.

^a^ The RCO construct definitions were adapted from Safran et al.^5^

^b^ Mix of social and task-oriented communication: conversations incorporate topics that are specific to work tasks as well as those that are personal and social.^5^

^c^ Mix of rich and lean communication: face-to-face discourse around areas of uncertainty or conflict and use of lean communication methods (eg, EHR) for simple or straightforward topics.^5^

^d^ Trust: psychological safety with colleagues and the belief that colleagues are committed and able to contribute to work.^5^

^e^ Mutual respect: the perception that behavior throughout an organization is rooted in integrity, appreciation, and thoughtfulness for others.^5^

^f^ Mindfulness: self-awareness and understanding of others in the organization.^5^

^g^ Heedful interrelating: interactions are guided by understandings of how one’s own contributions as well as those of others contribute to work tasks and organizational goals.^5^

### EHR Association With Communication

#### EHR as the Predominant Communication Modality in Health Care

Many physicians reported that the EHR has become the primary mode of communication between clinicians. Interviewees described core EHR communication as in-basket, an encrypted instant messaging portal that enables physician communication with patients and other care team members, and the clinical note, a repository of patient information. In addition, EHR communication included referrals, prescriptions, laboratory test results, and other domains.

One physician highlighted, “pretty much the majority of communication between my office staff is done through the EHR” (physician 8; 2018). Furthermore, several physicians reported that communication mediated by the EHR has weakened the relationships between members of care teams: “[the EHR] takes away a lot of face-to-face time... it just seems like the culture of the place that you work, you know everyone’s name based on the notes that they write, but you have no idea who that person is” (physician 17; 2018).

Many physicians noted that the EHR can be suboptimal for the task of team communication, in part because legal and billing requirements overwhelm the relevant communicative information. Some physicians reported that important information in a note could potentially be distilled, but to address all billing and legal requirements, they must include information beyond what another clinician would need to understand the clinical case.

Many physicians reported receiving EHR-related messages over a variety of platforms: in-basket, email, and text. Physicians reported that this can increase the challenge of communication between care team members. One interviewee likened managing patient care communication over multiple modalities to driving a car before the development of stoplights: “Some of my colleagues text; some of them send it in… email; some of them send it as Epic provider-to-provider messages. What a mess… there’s no sort of manners and rules. Right? Sort of like… before they developed stoplights, and there were starting to be more and more cars. Right? Man, this is nuts. It’s like, ‘Who’s going first. Who’s talking to who?’” (physician 21; 2022).

#### EHR in Task-Oriented and Social Communication

Several interviewees reported that EHR dependence has increased the ease of task-oriented communication, which “allows team members to function more efficiently together” (physician 23; 2018) and can support coordination of patient care. However, physicians noted that the EHR decreased the ease of communicating in person by increasing the threshold for connection and serving as a barrier to humanistic interactions: “in some ways it’s handicapped us a little bit because we rely so much on electronic communication with other providers… there’s a much higher threshold to pick up the phone and call” (physician 9; 2018). Some physicians shared that the EHR may also impair social communication by eliminating time available for physicians to interact in person with patients and other care team members.

Overall, physicians reported that EHR-driven barriers to humanistic interactions can cause them to lose sight of the shared purpose of a care team: “We are all working as part of the same team, but when we all do that exclusively through an EHR interface, and we lose that human touch that makes people even want to work on teams in the first place. Tasks become just tasks, and it is annoying. You start to lose sight of, ‘Oh yeah, this is for the patient. We are all in this together’” (physician 23; 2018).

#### EHR in Lean (Efficient) and Rich Communication

Many physicians felt that the EHR supported communication on well-defined topics. Physicians specifically cited the EHR in-basket as a tool to communicate efficiently with colleagues about scheduling, administrative, and simple clinical issues.

Despite these benefits, several interviewees also reported that the EHR has become the preferred modality for airing disagreements. Through the lens of the RCO, the EHR has harmed rich communication, which concerns emotionally charged issues. For example, an interviewee reported that other clinicians “would document petty, kind of nasty comments in the EHR about residents,” and noted that the discussion of such disagreements over EHR instead of in person can create “a sense of betrayal” (physician 2; 2018). Another interviewee hypothesized that the EHR has become the predominant modality to address emotionally charged topics because it is “the path of least resistance” and enables conflict avoidance (physician 1; 2018). The Figure summarizes the participant-reported modes by which task-oriented, social, lean, and rich communications are currently occurring and the modalities in which participants would prefer communication around these areas to occur.

**Figure.**
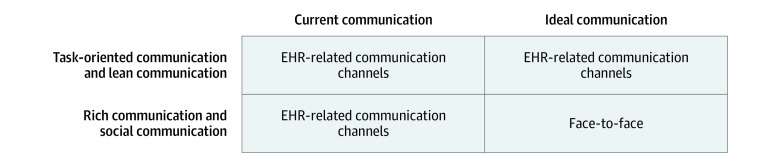
Modes of Communication by Health Care Workers EHR indicates electronic health record.

### EHR Association With Team Function

#### Medical-Legal Characteristics and Clinician Trust

Some interviewees expressed concern that the medical-legal characteristics of EHR may be harming team cohesion. One physician reported that the pressure to protect oneself from potential litigation may incentivize members of the care team to “put people under the bus” in EHR documentation: “I think the most frustrating part is like when folks are so afraid of litigation… they would say, ‘I paged M.D. for this patient, the doctor didn’t do anything.’” (physician 12; 2018). Using the RCO framework, tension arising from EHR medical-legal emphasis can be understood to harm trust, undermining physicians’ perceived psychological safety.

#### EHR and Mutual Respect Between Physicians and Other Clinicians

Physicians reported that the EHR has introduced a new set of tasks and responsibilities that are not always clearly defined. Some interviewees felt that this tension around EHR roles has largely eroded relationships between physicians and other clinicians, with physicians perceiving other clinicians as EHR managers rather than care team members: “I think what nursing has devolved into, as a nonnurse observer, is massaging the electronic medical record. The delivery of care to the patient… that’s completely been subsumed in documentation requirements” (physician 15; 2018). Overall, EHR role confusion can be understood as a challenge to the RCO construct of mutual respect.

#### Lack of Norms Around EHR Communication

Physicians mentioned that a lack of established norms around EHR communication can introduce frustration into care team interactions. Other clinicians may send extraneous messages due to the increased accessibility enabled by the in-basket; this can dilute the EHR in-basket as an efficient communication channel.

Physicians reported that clinician expectations around EHR-related communication can exacerbate physician workload and harm team relations. Physicians found that other care team members often expect instantaneous responses to communication: “now that I can place an order from anywhere, everyone assumes I can place an order from anywhere, and expects me to do so anywhere, anytime” (physician 21; 2022). Another physician added that the increased accessibility enabled by the EHR has led to “a breakdown” in what other members of the care team consider to be “an urgent issue” (physician 22; 2022).

This area of conflict can be understood through the RCO framework as a challenge to mindfulness and heedful interrelating. The rapid implementation of novel EHR-related communication platforms was not accompanied by best practice guidelines, making it a challenge for care team members to conscientiously engage with their colleagues.

### EHR Association With Team Member Well-being

#### EHR and Physician Joy

Physicians reported that in-person communication and connection with patients and colleagues is an essential source of physician joy: “… I can rush through my day, or I can find that connecting with that patient or even the nurse, or anyone… can just be enjoyable. Otherwise, we are just, you know, kind of going about our business, not interacting” (physician 24; 2018). Many physicians highlighted that the EHR has minimized this relational aspect of the physician role, thereby diminishing “physician happiness, obviously” (physician 1; 2018). Through the RCO lens, minimization of areas of joy acts as a barrier to the construct of heedful interrelating, as this aspect of the EHR impact challenges physicians’ sense of purpose in their work.

#### Physician Frustration With the EHR

Many physicians reported that onerous EHR design can cause distress: “everything in the EMR takes 100 clicks to do… each click is like a little, little stab wound” (physician 3; 2018). Interviewees highlighted that physician distress related to EHR functionality can harm interactions with patients and the care team: “I’m angry at the EHR a lot, so, and I express my frustration often and verbally, which nurses will hear, patients will sometimes hear. They can tell when you’re frustrated with the system… it bleeds over into your interactions… by ripple effect it’s just going to… create a toxic environment for everybody.” (physician 7; 2018). Physician frustration over EHR functionality challenges the RCO construct of heedful interrelating, because this area of EHR impact can create frustration and anger that seeps into physicians’ interactions with their colleagues.

## Discussion

Overall, our analysis guided by RCO suggests that the EHR is intertwined in every aspect of health care team dynamics, with implications for communication, team function, and team member well-being (eFigure 2 in Supplement 1). Interviewees expressed that the EHR has improved lean and task-related communication well suited to completing simple, uncomplicated tasks. However, the EHR has limited rich and social communication required to build relationships, solve complex clinical problems, and navigate conflict. Physicians perceived that the EHR negatively impacts team function by amplifying disagreement and introducing conflict originating in medical-legal pressure and undefined communication norms for the EHR. In addition, physician distress about the EHR may affect physician interactions with the team, injuring team member well-being. Our results suggest that the EHR supports looser forms of interprofessional work, such as networking and coordination, at the expense of more intense collaboration and teamwork.^32,33^

Our results emphasize the need for in-person conversation, both to build relationships via social connection and to address areas of conflict. Social interaction, minimized by the EHR, is key for the development of workplace trust, meaning, and satisfaction.^34,35,36,37,38,39,40,41^ Our results confirm past findings around the importance of face-to-face interaction in supporting physician well-being. Direct interaction may mediate EHR-related physician distress and enable physicians to build relationships and address conflict.^34,42^ Furthermore, the use of virtual team communication strategies that support rich and social communication, such as discussing conflicts over a video call or securing protected time for virtual social communication, may address some of the communication challenges identified in this analysis.^40,41,43^

Another major finding relates to the EHR interaction with team function. Overall, the EHR is introducing novel and unexperienced areas of conflict, via undefined roles, processes, and areas of mistrust.^44,45,46,47,48^ Since team function is a crucial aspect of patient care,^47^ considerations to address and support best practices of team functioning (eg, dedicating time to team formation) may need to be integrated into team-based care.^49,50,51,52^

Our final major finding centered around the EHR and individual physician well-being. Most studies investigating the association between team function and physician well-being have focused on the role that team function may play in physician well-being.^15^ Our study explores a reverse connection, suggesting that fostering physician well-being may be beneficial for team function and therefore may minimize unprofessional behaviors instigated by physician distress.^18,53,54,55,56^ This represents yet another rationale for system approaches to address burnout in health care.

This research addresses a major gap in the literature and has the potential to serve as a launching pad for future efforts to optimize care team function. Our findings suggest that understandings of optimal EHR use are needed and that the development and improvement of local work culture is critical and may have a greater influence on physician burnout than EHR improvements alone.^57,58^ Solutions focused on the cultivation of team connection and trust can be highly productive, even as the EHR continues to be used appropriately. For example, we suggest organizations support physicians in implementing small, structured peer-group discussions to enhance team function and individual well-being.^34,59,60^ Improving team stability may also enable teams to establish norms around face-to-face and EHR-related communication modalities.^16,61,62^

### Limitations

This study has limitations. Given the themes around physician relationships with other clinicians, the sole inclusion of physicians in this analysis is a limitation. Another limitation is that this analysis represents a secondary analysis of qualitative interview data. Thus, the interview guides were not specifically designed to address team members’ perceptions of the interplay between team relational dimensions, the EHR, and physician well-being. Nonetheless, these topics were discussed and emerged during the interviews. In addition, data used in this analysis were collected both before and during the COVID-19 pandemic. The COVID-19 pandemic was a period when both physician burnout increased and the use of EHR-based communication strategies expanded widely.^19,63^

## Conclusions

In this study, participants reported that the EHR supports task-oriented and efficient communication among team members to get work done and efficiently care for patients. However, findings of this study suggest that the EHR shifts attention away from the human needs of the care team necessary for developing relationships, building trust, and resolving conflicts that are foundational to patient care and principles of humanism in medicine. Future research can further investigate interventions to maintain humanism in the face of greater integration of health information technology in care delivery.

## Notes

Supplement 1. **eFigure 1.** Existing Understandings of the Interplay Between the EHR, Team Function, Team Communication, and Physician Well-being **eFigure 2.** Described Relationships Between the EHR, Team Communication, Team Function, and Physician Well-Being **eMethods.** Detailed Methods **eTable 1.** Study 1 Semi-Structured Interview Protocol **eTable 2.** Study 2 Semi-Structured Interview Protocol
eReferences

Supplement 2.
Data Sharing Statement
