# Supplementary material for: Perspectives on the Intersection of Electronic Health Records and Health Care Team Communication, Function, and Well-being
Source: JAMA Netw Open. 2023 May 12;6(5):e2313178. doi: 10.1001/jamanetworkopen.2023.13178 (PMC10182436; doi:10.1001/jamanetworkopen.2023.13178)
Supplement: Supplement 1. — eFigure 1. Existing Understandings of the Interplay Between the EHR, Team Function, Team Communication, and Physician Well-being eFigure 2. Described Relationships Between the EHR, Team Communication, Team Function, and Physician Well-Being eMethods. Detailed Methods eTable 1. Study 1 Semi-Structured Interview Protocol eTable 2. Study 2 Semi-Structured Interview Protocol eReferences [file jamanetwopen-e2313178-s001.pdf]

Supplementary Online Content

Amano A, Brown-Johnson CG, Winget M, et al. Perspectives on the intersection of electronic health records and health care team communication, function, and well-being. *JAMA Netw Open*. 2023;6(5):e2313178. doi:10.1001/jamanetworkopen.2023.13178

eFigure 1. Existing Understandings of the Interplay Between the EHR, Team Function, Team Communication, and Physician Well-being

eFigure 2. Described Negative Influences Between the EHR, Team Communication, Team Function, and Physician Well-being

eMethods. Detailed Methods

eTable 1. Study 1 Semi-Structured Interview Protocol

eTable 2. Study 2 Semi-Structured Interview Protocol

eReferences

This supplementary material has been provided by the authors to give readers additional information about their work.

© 2023 Amano A et al. *JAMA Network Open.*

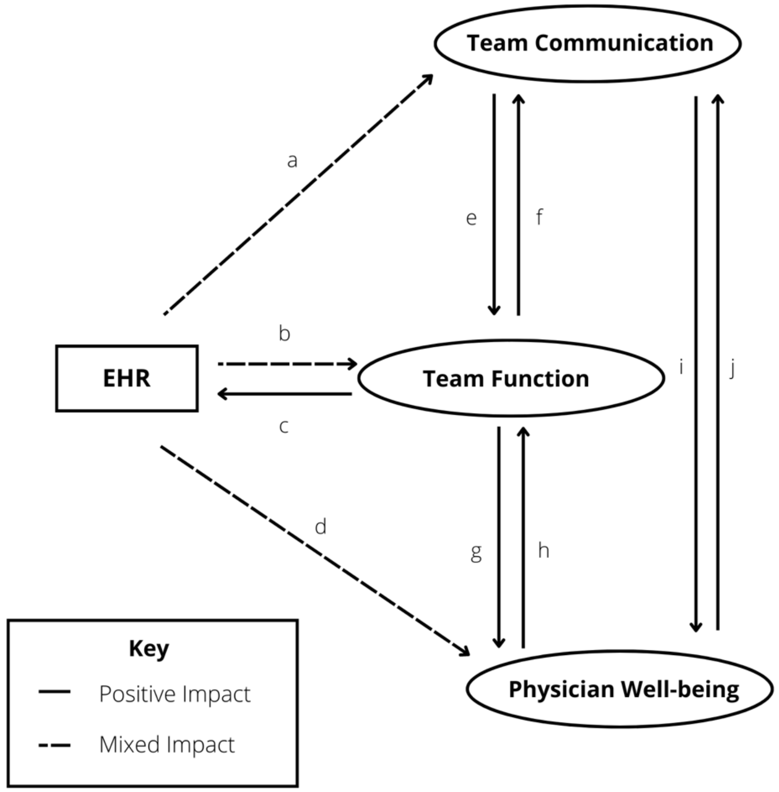

eFigure 1. Existing Understandings of the Interplay Between the EHR, Team Function, Team Communication, and Physician Well-being

Notes:

[a] The EHR has had mixed impacts on team communication.[1–3]

[b] The EHR has had mixed impacts on team function.[2–4]

[c] Team function can support uptake and usage of the EHR.[5,6]

[d] The EHR has had mixed impacts on physician well-being.[1,7–10]

[e] Team communication positively impacts team function.[11–14]

[f] Team function positively impacts team communication.[14–19]

[g] Team function positively impacts physician well-being.[20,21]

[h] Physician well-being positively impacts team function.[22]

[i] Team communication positively impacts physician well-being.[23,24]

[j] Physician well-being positively impacts team communication.[25,26]

© 2023 Amano A et al. *JAMA Network Open.*

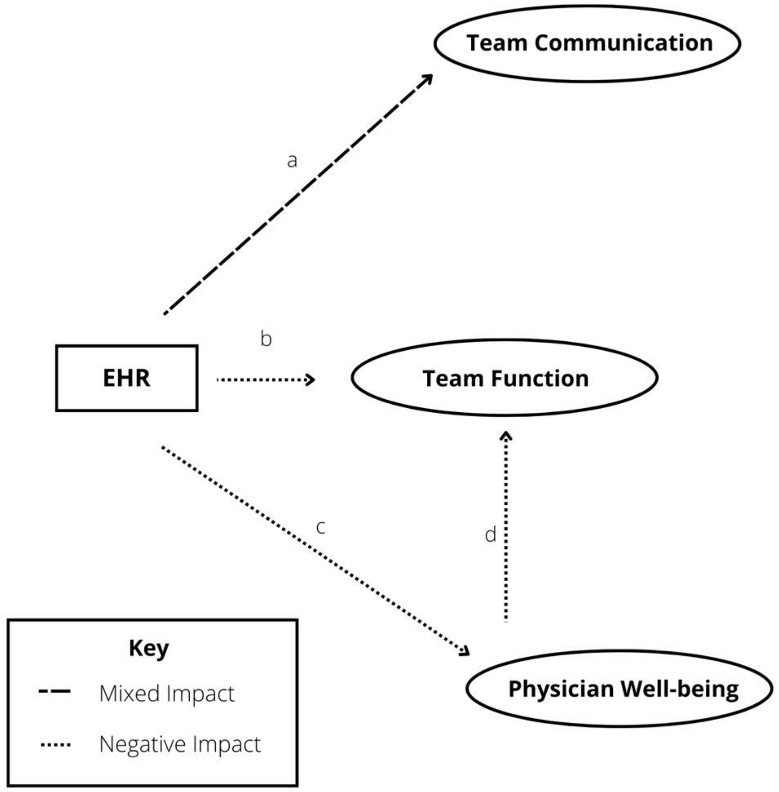

eFigure 2. Described Negative Influences Between the EHR, Team Communication, Team Function, and Physician Well-being

Notes:

[a] The EHR supported some aspects of team communication (task-oriented and lean) while hurting others (social and rich). Understandings of the specific impacts of the EHR on each component of team communication are novel.

[b] The EHR harmed team function by introducing novel areas of conflict. This finding re-enforced pre-existing literature.

[c] The EHR physician well-being by removing sources of physician joy and introducing novel sources of friction. This finding re-enforced pre-existing literature that has identified that the EHR has both positive and negative impacts on physician well-being.

[d] When physician well-being harmed it was found to also harm team function. This relationship is novel.

© 2023 Amano A et al. *JAMA Network Open.*

eMethods. Detailed Methods

Study 1 and 2 EHR Information

EHR systems included CERNER and EPIC.

Study 2 Qualitative Data Collection Information

We conducted our study in two healthcare organizations in Northern California—an academic medical center (Site A) and a community hospital (Site B). To cover the broadest viewpoint of physician distress, we recruited from a variety of ambulatory and non-proceduralist specialties. Interviews were completed via video (Zoom) or phone call between February 28 to April 21, 2022.

Participant Eligibility and Recruitment

To ensure participants had meaningful exposure to the EHR, physicians with an average of at least 8 weekly hours of scheduled patient time were included in this assessment. Participants were recruited from a wide range of clinical areas, including Primary Care (Internal Medicine and Family Medicine), Hematology/Oncology, Psychiatry, Neurology, Dermatology, Endocrinology, Nephrology, Rheumatology, Infectious Diseases, and Allergy/Immunology. The participant population was stratified to reflect the size of each clinical area: 4 physicians practicing in Primary Care, the largest clinical area, and 1- 2 physicians in each of the remaining clinical areas were recruited for participation.

Sampling Strategy

Recruitment preserved participant anonymity. A member of the research team sent an e-mail to a random sample of potential participants inviting them to participate in the study and stating the study purpose: to learn more about physicians' perspectives regarding helpful and challenging aspects of the EHR during the COVID-19 pandemic. To protect data collection, participants' contact information was stored in a HIPAA compliant data storage platform (Box). Interviews were scheduled and conducted by the two qualitative researchers on our research team (A.A., C.B.J.).

© 2023 Amano A et al. *JAMA Network Open.*

eTable 1. Study 1 Semi-Structured Interview Protocol

Topic area	Prompt(s)
Appreciative inquiry	How is the EHR helping you? In patient care? In saving time? In fulfillment as a physician?
Opening interview question	Tell me about how you use the EHR in your daily work.
Positive aspects of EHR	How is the EHR helping you? In patient care? In saving time? In fulfillment as a physician?
Emotion/ Trigger Source	I now would like to talk about your negative experiences with using the EHR, and more specifically times where you have actually had an emotionally negative reaction to using the EHR.<br>1. Have you felt a negative emotion with an EHR activity over the past 2 weeks ("Think about something in the last week that really bothered you about your interaction with the EHR" – What emotion were you feeling? OR What aspect of the EHR increases your sense of stress as a physician?<br>2. Identify the trigger activity that led to that negative emotion – what activity were you doing? Please be as specific as possible.<br>3. Why did you have to do this activity? Who/what required it?<br>4. Why do you think physicians, rather than others, are asked to do this activity?<br>5. Who else could have completed this activity?
Validity of Information Entered	In responding to documentation requirements, physicians may feel inclined to document things that were not actually performed, completed or that are invalid. Have you had to do that? If yes, can you provide an example of such a situation?<br><br>These draft questions below are more specific and possibly more emotional. Consider whether any of the questions are more effective than others for this purpose.<br>6. How often do you verify information (e.g. medicine reconciliation, allergies) without actually performing the task?<br>7. How does this make you feel?<br>a. What is driving you to complete this task without validation?<br>b. Possible prompts:<br>i. To be expedient,<br>ii. answer not relevant to patient care,<br>iii. have to answer to complete documentation requirements.<br>8. Do you always perform the review of systems that you document as having completed?<br>a. If not, why do you document the review of systems in this manner?<br>b. How does this make you feel?<br>9. Do you always perform the physical exam that you document as having completed?<br>a. If not, why do you document the exam in this manner?<br>b. How does this make you feel?

© 2023 Amano A et al. *JAMA Network Open.*

	10. Why did you feel the need to report unvalidated or inaccurate information in your documentation?<br>11. How often are you documenting questionable information?<br>a. How does this make you feel?<br>b. What is driving you to document in this manner?
Meaningfulness of Activities	Some physicians are reporting less meaning and purpose in their activities. I will now ask you questions examining whether the EHR tasks are exacerbating this problem for you.<br><br>12. What is the impact of EHR documentation, in general, on your professional life/meaning, e.g., how is this process affecting your work?<br>13. Although some EHR tasks can improve patient care, some may take you away from other more important activities. Do you feel this way?<br>a. If YES:<br>i. Is the time you spend documenting in the EHR preventing you from spending time with your patients?<br>ii. Are the EHR tasks affecting your time/motivation to learn/read about your patient's disease, i.e., are these tasks affecting your learning?<br>iii. How does this make you feel?<br>14. Are there any aspects of the EHR makes you feel that you are doing the wrong kind of work? Or question being a physician?
Helplessness	I will now transition to what you have done with these experiences with the EHR. The following questions will address whether negative experiences are brought to the awareness of the administration.<br><br>15. Have you revealed your opinion about your experiences with the EHR? If so, to whom? If not, why not?<br>16. Have you had errors or near misses in your practice?<br>a. If yes, did you report these to the administration/risk authority or your superiors?<br>b. If not, why not?<br>17. Have you mentioned any of these concerns to the administration or your superiors?<br>a. If so, to whom?<br>b. If not, why not?<br>18. Do you feel comfortable bringing these feelings to the hospital administration?<br>a. If not, why?<br>19. Do you think there could be negative repercussions of sharing your experience/opinion?
	I will ask about the existence or effectiveness of current mechanisms for physicians to reveal their experiences, viewpoints and recommendations to administration.<br><br>20. Do you feel that you have a meaningful voice within the institution? Are physician voices valued by the administration?

© 2023 Amano A et al. *JAMA Network Open.*

	a. If yes, how is your opinion being heard?<br>b. If no, what recommendations do you have for the institution regarding awareness and usefulness of physician input and experience?<br>21. If you were to be able to have an impact on this type of activity – e.g., make it more effective, less difficult, etc., how do you think you would like to share your recommendation with the administration, e.g., in person/ focus groups/ electronically?

© 2023 Amano A et al. *JAMA Network Open.*

eTable 2. Study 2 Semi-Structured Interview Protocol

Topic area	Prompt(s)
Appreciative inquiry	What do you personally find most gratifying about your work as a physician?
Opening interview question	In general, how would you describe your experience with the EHR?<br>How has it changed during the pandemic?
Positive aspects of EHR	What are EHR tasks that have been beneficial to you over the past 2 weeks?<br>How does the EHR positively impact or facilitate your ability to provide patient care?<br>How has it changed during the pandemic?
Negative aspects of the EHR	What are EHR tasks that have been burdensome over the past 2 weeks?<br>What are some factors contributing to these tasks being burdensome?<br>How does the EHR negatively impact or impede your ability to provide patient care?<br>How has it changed during the pandemic?<br>In an average week, how many hours do you spend on EHR related activities during personal time on nights and weekends?<br>In your opinion, how much of that time is due to EHR inefficiencies and how much is due to excessive clinical work-load during usual work time?
Strategies to address EHR challenges	What strategies have been helpful in reducing the burden of EHR-related activities?<br>What are some improvement interventions that have helped reduce your burden of EHR-related activities?<br>What factors limit the availability or impact of EHR burden reduction efforts in your work area? (eg: time, lack of knowledge etc.)<br>How has it changed during the pandemic?<br>What are some EHR burden reduction efforts that you wish you had more of or could take more advantage of?<br>What interventions would you like to see to reduce the burden of EHR-related activities?

© 2023 Amano A et al. *JAMA Network Open.*

eReferences

1. Gardner RL, Cooper E, Haskell J, et al. Physician stress and burnout: the impact of health information technology. *Journal of the American Medical Informatics Association*. 2019;26(2):106-114. doi:10.1093/jamia/ocy145

2. Quinn M, Forman J, Harrod M, et al. Electronic health records, communication, and data sharing: challenges and opportunities for improving the diagnostic process. *Diagnosis*. 2019;6(3):241-248. doi:10.1515/dx-2018-0036

3. Janssen A, Robinson T, Brunner M, Harnett P, Museth KE, Shaw T. Multidisciplinary teams and ICT: a qualitative study exploring the use of technology and its impact on multidisciplinary team meetings. *BMC Health Serv Res*. 2018;18(1):444. doi:10.1186/s12913-018-3242-3

4. O'Malley AS, Draper K, Gourevitch R, Cross DA, Scholle SH. Electronic health records and support for primary care teamwork. *Journal of the American Medical Informatics Association*. 2015;22(2):426-434. doi:10.1093/jamia/ocu029

5. Gross AH, Leib RK, Tonachel A, et al. Teamwork and Electronic Health Record Implementation: A Case Study of Preserving Effective Communication and Mutual Trust in a Changing Environment. *J Oncol Pract*. 2016;12(11):1075-1083. doi:10.1200/JOP.2016.013649

6. Graetz I, Reed M, Shortell SM, Rundall TG, Bellows J, Hsu J. The Association between EHRs and Care Coordination Varies by Team Cohesion. *Health Serv Res*. 2014;49(1pt2):438-452. doi:10.1111/1475-6773.12136

7. Downing NL, Bates DW, Longhurst CA. Physician Burnout in the Electronic Health Record Era: Are We Ignoring the Real Cause? *Ann Intern Med*. 2018;169(1):50. doi:10.7326/M18-0139

8. Ashton M. Getting Rid of Stupid Stuff. *N Engl J Med*. 2018;379(19):1789-1791. doi:10.1056/NEJMp1809698

9. Dietsche E. Judy Faulkner asked the press to dig into physician burnout and EHR satisfaction. So we did. *MedCity News*. Published online March 7, 2019. https://medcitynews.com/2019/03/physician-burnout-ehr-satisfaction/

10. Skeff KM, Brown-Johnson CG, Asch SM, Zionts DL, Winget M, Kerem Y. Professional Behavior and Value Erosion: A Qualitative Study of Physicians and the Electronic Health Record. *Journal of Healthcare Management*. 2022;Publish Ahead of Print. doi:10.1097/JHM-D-21-00070

11. Hearn J, Higginson IJ. Do specialist palliative care teams improve outcomes for cancer patients? A systematic literature review. *Palliat Med*. 1998;12(5):317-332. doi:10.1191/026921698676226729

12. Weller J, Boyd M, Cumin D. Teams, tribes and patient safety: overcoming barriers to effective teamwork in healthcare. *Postgrad Med J*. 2014;90(1061):149-154. doi:10.1136/postgradmedj-2012-131168

13. Lingard L, Reznick R, Espin S, Regehr G, DeVito I. Team Communications in the Operating Room: Talk Patterns, Sites of Tension, and Implications for Novices. *Academic Medicine*. 2002;77(3). https://journals.lww.com/academicmedicine/Fulltext/2002/03000/Team_Communications_in_the_Operating_Room__Talk.13.aspx

© 2023 Amano A et al. *JAMA Network Open.*

14. Rosen MA, DiazGranados D, Dietz AS, et al. Teamwork in healthcare: Key discoveries enabling safer, high-quality care. *Am Psychol*. 2018;73(4):433-450. doi:10.1037/amp0000298

15. Sargeant J, Loney E, Murphy G. Effective interprofessional teams: "Contact is not enough" to build a team. *Journal of Continuing Education in the Health Professions*. 2008;28(4):228-234. doi:10.1002/chp.189

16. Temkin-Greener H, Gross D, Kunitz SJ, Mukamel D. Measuring Interdisciplinary Team Performance in a Long-Term Care Setting. *Medical Care*. 2004;42(5):472-481. doi:10.1097/01.mlr.0000124306.28397.e2

17. Davis P, Man P, Cave A, McBennett S, Cook D. Use of focus groups to assess the educational needs of the primary care physician for the management of asthma. *Med Educ*. 2000;34(12):987-993. doi:10.1046/j.1365-2923.2000.00685.x

18. Apker J, Propp KM, Zabava Ford WS, Hofmeister N. Collaboration, Credibility, Compassion, and Coordination: Professional Nurse Communication Skill Sets in Health Care Team Interactions. *Journal of Professional Nursing*. 2006;22(3):180-189. doi:10.1016/j.profnurs.2006.03.002

19. Lingard L, Reznick R, DeVito I, Espin S. Forming professional identities on the health care team: discursive constructions of the 'other' in the operating room: Forming professional identities. *Medical Education*. 2002;36(8):728-734. doi:10.1046/j.1365-2923.2002.01271.x

20. Willard-Grace R, Hessler D, Rogers E, Dube K, Bodenheimer T, Grumbach K. Team Structure and Culture Are Associated With Lower Burnout in Primary Care. *The Journal of the American Board of Family Medicine*. 2014;27(2):229-238. doi:10.3122/jabfm.2014.02.130215

21. Linzer M, Poplau S, Brown R, et al. Do Work Condition Interventions Affect Quality and Errors in Primary Care? Results from the Healthy Work Place Study. *J GEN INTERN MED*. 2017;32(1):56-61. doi:10.1007/s11606-016-3856-2

22. Welp A, Meier LL, Manser T. The interplay between teamwork, clinicians' emotional exhaustion, and clinician-rated patient safety: a longitudinal study. *Crit Care*. 2016;20(1):110. doi:10.1186/s13054-016-1282-9

23. West CP, Dyrbye LN, Shanafelt TD. Physician burnout: contributors, consequences and solutions. *J Intern Med*. 2018;283(6):516-529. doi:10.1111/joim.12752

24. DeChant PF, Acs A, Rhee KB, et al. Effect of Organization-Directed Workplace Interventions on Physician Burnout: A Systematic Review. *Mayo Clinic Proceedings: Innovations, Quality & Outcomes*. 2019;3(4):384-408. doi:10.1016/j.mayocpiqo.2019.07.006

25. Quenot JP, Rigaud JP, Prin S, et al. Suffering among carers working in critical care can be reduced by an intensive communication strategy on end-of-life practices. *Intensive Care Med*. 2012;38(1):55-61. doi:10.1007/s00134-011-2413-z

26. Vermeir P, Blot S, Degroote S, et al. Communication satisfaction and job satisfaction among critical care nurses and their impact on burnout and intention to leave: A questionnaire study. *Intensive and Critical Care Nursing*. 2018;48:21-27. doi:10.1016/j.iccn.2018.07.001

© 2023 Amano A et al. *JAMA Network Open.*
