# Supplementary material for: Perspectives on the Intersection of Electronic Health Records and Health Care Team Communication, Function, and Well-being
Source: JAMA Netw Open. 2023 May 12;6(5):e2313178. doi: 10.1001/jamanetworkopen.2023.13178 (PMC10182436; doi:10.1001/jamanetworkopen.2023.13178)
Supplement: Supplement 2. — Data Sharing Statement [file jamanetwopen-e2313178-s002.pdf]

Data Sharing Statement

Amano. Perspectives on the Intersection of Electronic Health Records and Health Care Team Communication, Function, and Well-being. *JAMA Netw Open*. Published May 12, 2023. doi:10.1001/jamanetworkopen.2023.13178

Data
Data available: No

Additional Information
Explanation for why data not available: not applicable
